# miR-455-5p promotes cell growth and invasion by targeting SOCO3 in non-small cell lung cancer

**DOI:** 10.18632/oncotarget.22565

**Published:** 2017-11-20

**Authors:** Junfeng Wang, Yanbo Wang, Dawei Sun, Jianlong Bu, Fenghai Ren, Benkun Liu, Shibo Zhang, Zigeng Xu, Sainan Pang, Shidong Xu

**Affiliations:** ^1^ The Department of Thoracic Surgery, Harbin Medical University Cancer Hospital, Harbin 150081, China

**Keywords:** miR-455-5p, SOCO3, invasion, non-small cell lung cancers

## Abstract

Non-small cell lung cancer (NSCLC) is the most common type of lung cancer. miR-455-5p has increased expression and the ability to promote tumorigenesis in certain cancers. However, the role of miR-455-5p in NSCLC has not been sufficiently investigated. SOCS3 (suppressor of cytokine signaling 3), an important tumor suppressor, is often aberrantly inactivated in various tumors, but it is currently unclear whether SOCO3 is a target of miR-455-5p. In the present study, we investigated the role of miR-455-5p in NSCLC. We found that the expression of miR-455-5p was up-regulated in NSCLC tumor tissues compared to corresponding noncancerous tissues, and its expression was correlated with metastasis and tumor node metastasis in NSCLC tissue. We then showed that miR-455-5p promoted migration, invasion and proliferation in NSCLC cell lines. Additionally, we also found that SOCS3 was the direct target gene of miR-455-5p. Consistently, the expression of SOCS3 was negatively correlated with the expression of miR-455-5p in NSCLC tissues. We further show that aberrant miR-455-5p expression is partially controlled by activated ERK signaling in NSCLC. Therefore, miR-455-5p could enhance the growth and metastasis of NSCLC by inhibiting SOCS3, thus providing a potential molecular therapeutic target for the treatment of NSCLC patients.

## INTRODUCTION

Lung cancer becomes a main reason of cancer mortality throughout the world [1, 2]. Non-small cell lung cancer (NSCLC) is a major type cancer. NSCLC with metastasis is the primary cause of lung cancer mortality [3–5]. Thus, exploring the mechanisms of NSCLC tumorigenesis could be very helpful for NSCLC treatment through identifying effective therapies.

It is well-known that microRNAs (miRNAs) do not encode protein sequences, but negatively control mRNA stability and/or inhibit mRNA translation [6–8]. They can play important roles in various stages of tumorigenesis and tumor development [9]. Depending on the different target genes, miRNAs can promote or inhibit tumorigenesis [10, 11]. Accumulating reports indicated miR-455-5p is critical for many cancers. For example, miR-455-5p expression levels correlated with clinicopathological features and thus served as a prognostic and diagnostic biomarker in endometrial serous adenocarcinomas [12], basal cell carcinoma [13], laryngeal cancer [14], and hepatocellular adenoma [15]. It has been reported that miR-455-5p promoted melanoma metastasis by inhibiting CPEB1 [16]. However, the function of miR-455-5p in NSCLC is currently unknown.

In this study, we explored the function of miR-455-5p in NSCLC development. Our results showed that miR-455-5p promotes tumor growth and metastasis through inhibiting SOCS3 in NSCLC.

## RESULTS

### miR-455-5p is up-regulated in human NSCLC samples and correlated to NSCLC progression

**Figure 1:**
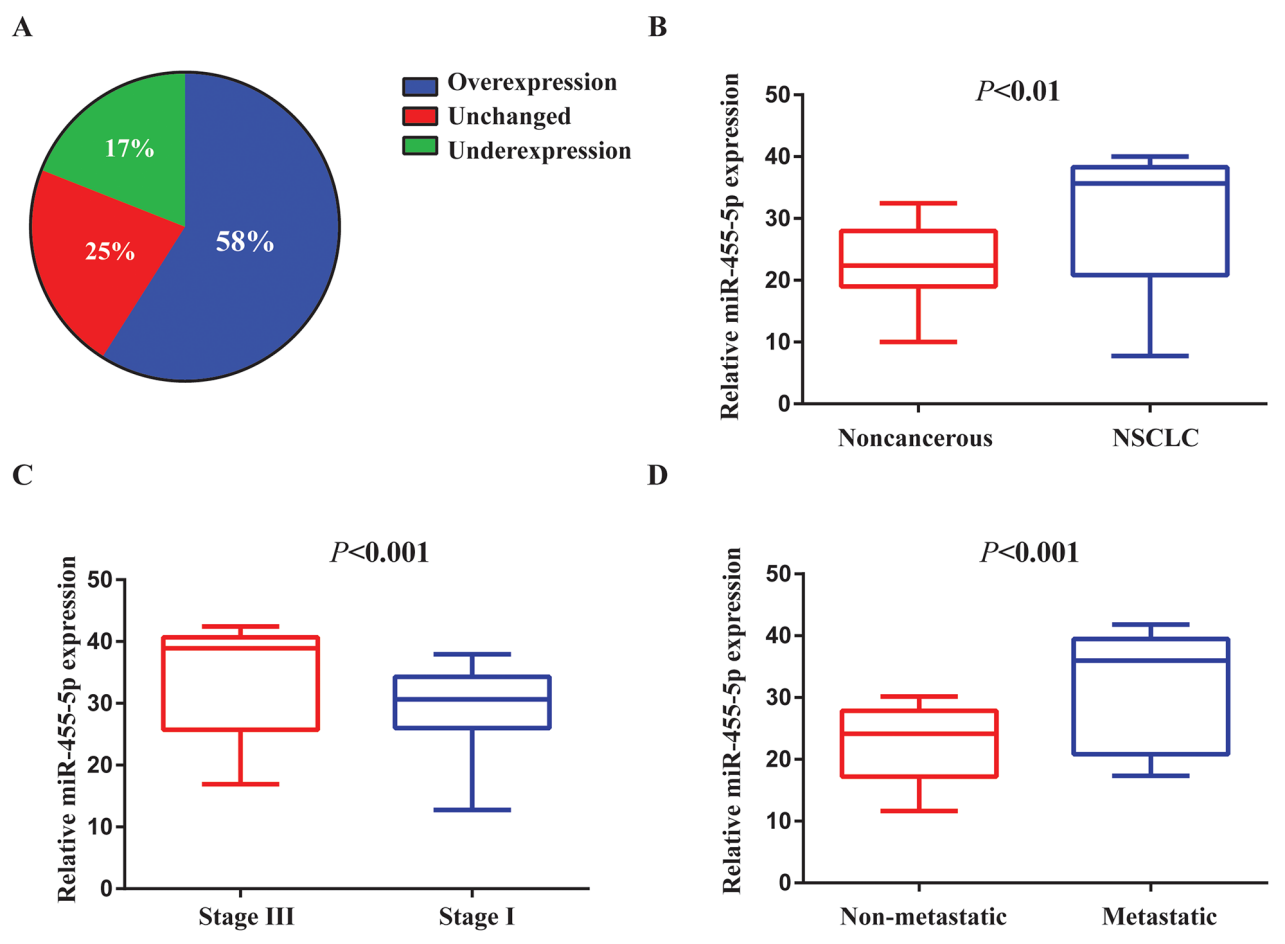
miR-455-5p is up-regulated in human NSCLC tissues and associated with NSCLC progression **(A)** The expression levels of miR-455-5p in 79 paired NSCLC and corresponding noncancerous tissues were measured by TaqMan real-time PCR and normalized against an endogenous U6 RNA control. **(B)** The expression of miR-455-5p was overexpressed in NSCLC tissues compared with the noncancerous tissues. **(C)** miR-455-5p expression was detected in different clinical stages of NSCLC. **(D)** The up-regulation of miR-455-5p in NSCLC was associated with tumor metastasis; the patients were classified into tumor metastasis negative and positive groups (lymph nodemetastasis and/ or distal metastasis) Error bars represent SEM. The qRT-PCR were analyzed and shown as relative miR-455-5p levels of the Ct (cycle threshold) values. The statistical analysis was performed using paired *t* test (B) and Student’s *t* test (C) and (D).

To find out the function of miR-455-5p in NSCLC cells, we firstly detected miR-455-5p expressionin 79 pairs of human NSCLC tissues and their comparable nontumor tissue. miR-455-5p expression was notably up-regulated in tumor tissues (58%) compared with the controls (Figure 1A and 1B). The levels of miR-455-5p were increased in advanced lung cancer (stage III, *n* = 46) to beginning stages (stage I, *n* = 33) (Figure 1C). Moreover, miR-455-5p expression was up-regulated in metastatic NSCLC (*n* = 50) compared to NSCLC that non-spread (*n* = 29) (Figure 1D).

### SOCS3 is a target of miR-455-5p

**Figure 2:**
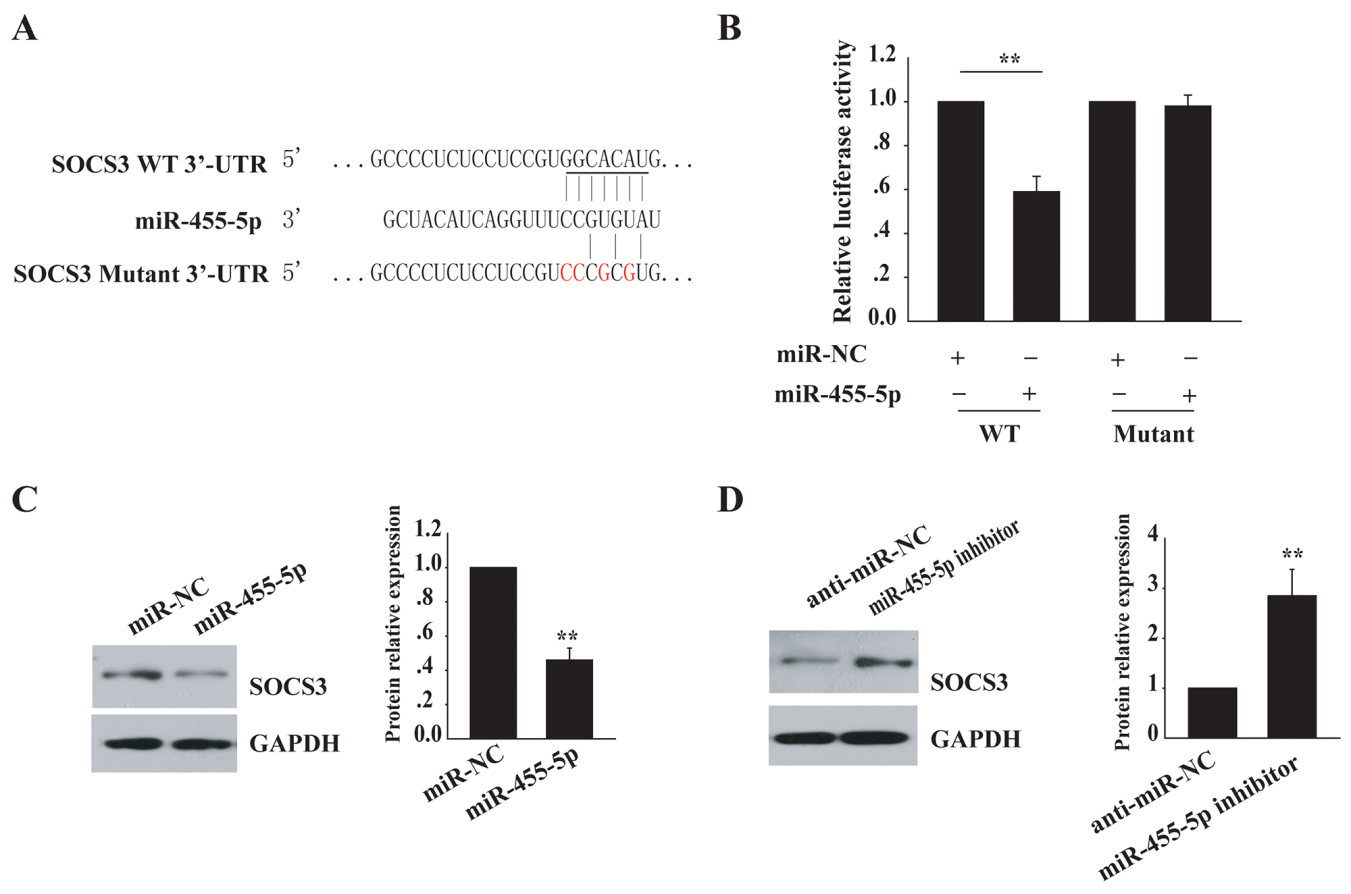
SOCS3 is a target of miR-455-5p **(A)** SOCS3is predicted to be a target of miR-455-5p. The sequences of the putative miR-455-5p binding sites in wild type and mutant (red) SOCS3-3′UTR. **(B)** SOCS3 3′-UTR luciferase reporter assays in 293T cells. **(C)** Western blot analysis of SOCS3 protein levels in miR-455-5p mimics transfected H460 cells. **(D)** Western blot analysis of SOCS3 protein levels in anti- miR-455-5p-transfected H1299 cells. Error bars represent SD. ^**^, P < 0.01. Results shown are representative of three independent experiments.

Using the computational prediction program TargetScan, we found that suppressor of cytokine signaling (SOCS) 3, which plays a role in the feedback inhibition of JAK/STAT signaling [17–19], was a supposed target of miR-455-5p (Figure 2A). To explore whether miR-455-5pregulates SOCS3, the wild-type SOCS3 3′-UTR was cloned, and transfected the reporters together with control RNA or miR-455-5p mimics. Co-transfection of miR-455-5p mimics notably blocked (1.7 fold) the wild-type reporter activity, in contrast, the mutant reporter was almost not affected (Figure 2B), suggesting that SOCS3 is a target of miR-455-5p.

Then, we compared miR-455-5p expression in a normal lung cell line and NSCLC cell lines. qRT-PCR was employed to detect its expression in several cell lines, including H460, SK-MES-1, SPCA-1, A549, and H1299, and the normal lung cell line 16HBE. miR-455-5p was highly expressed in all NSCLC cell lines examined. In contrast, its expression was low in the normal lung cell line 16HBE (Supplementary Figure 1). We transfected miR-455-5pmimics in H460 cells, a decreased endogenous level of miR-455-5p, and tested the endogenous SOCS3 expression by Western blot assay. The level of SOCS3 protein was inhibited (2 fold) in cells with miR-455-5pmimic transfection but no change in control cells (Figure 2C). However, inhibition of miR-455-5p by anti-miR-455-5p in H1299 cells, an increased endogenous miR-455-5p expression, resulted in enhanced SOCS3 expression (3 fold) (Figure 2D). These results show that SOCS3 is a direct target of miR-455-5p.

### miR-455-5p promotes tumorigenesis by targeting SOCS3 in NSCLC

**Figure 3:**
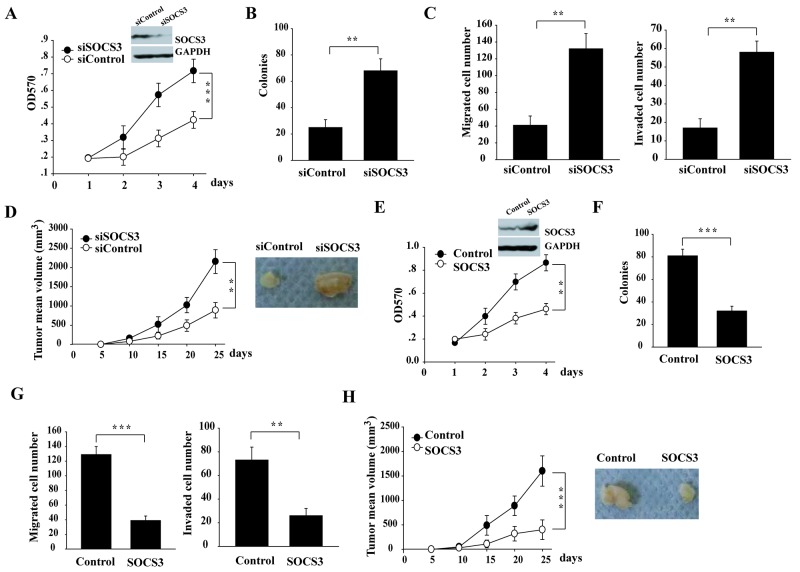
miR-455-5p promotes NSCLC tumorigenesis via targeting SOCS3 **(A-D)** RNAi knockdown of SOCS3 in H460 cells promoted the cell proliferation (A), soft-agar colony formation assays (B), Transwell cell migration and invasion (C), and xenograft tumor growth in nude mice (D) **(E-H)**, ectopic expression of SOCS3 in H1299 cells decreased the cell proliferation (E), soft-agar colony formation assays (F), Transwell cell migration and invasion (G), and xenograft tumor growth in nude mice (H). Error bars represent SD. ^**^, P < 0.01; ^***^, P < 0.001. Results shown are representative of three independent experiments.

As a previous study showed that miR-455-5p inhibits the tumor suppressor gene CPEB1 expression to facilitate melanoma metastasis [16], we next test whether the targeting of SOCS3 by miR-455-5p is important for NSCLC. We found that SOCS3 knockdown in H460 cells with higher level of SOCS3, significantly promoted cell proliferation (Figure 3A, Supplementary Figure 2), anchorage-independent growth (3 fold; Figure 3B), cell migration and invasion (Figure 3C), and tumor growth *in vivo* (Figure 3D), suggesting a tumor-suppressive role of SOCS3 in NSCLC cells. Additionally, overexpression of Flag-SOCS3 in H1299 cells with lower SOCS3 level, dramatically reduced cell proliferation, colony formation, cell migration and invasion, and tumor growth *in vivo* (Figure 3E-3H). Thus, SOCS3 plays a tumor-suppressive function in NSCLC cells.

**Figure 4:**
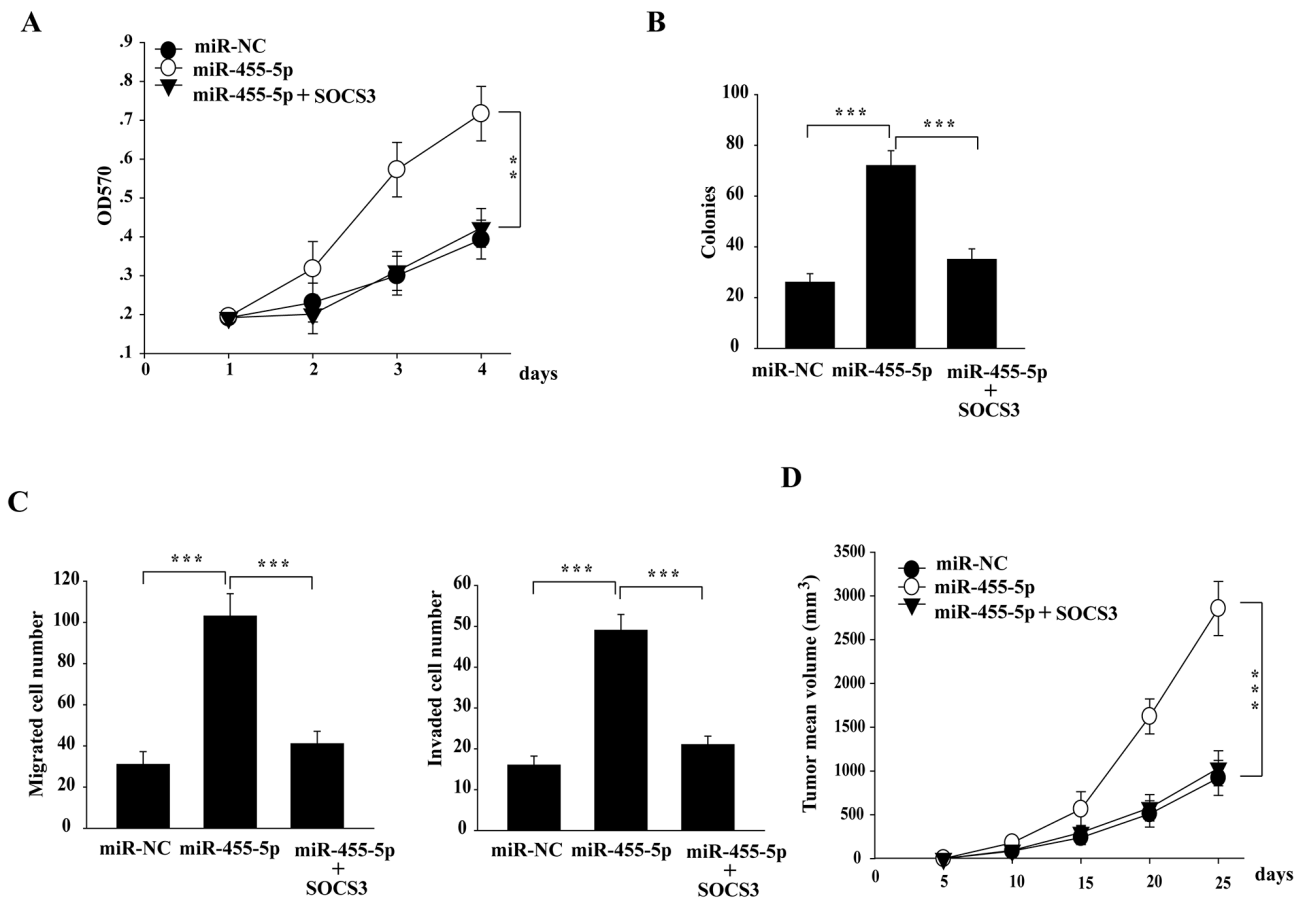
The miR-455-5p-SOCS3 axis is of functional importance in regulating tumorigenesis in NSCLC cells **(A-D)** ectopic expression of SOCS3 in miR-455-5p mimics-transfected H460 cells significantly attenuated the effect of miR-455-5p on the cell proliferation (A), soft-agar colony formation assays (B), Transwell cell migration and invasion (C), and xenograft tumor growth in nude mice (D). Error bars represent SD. ^**^, P < 0.01; ^***^, P < 0.001. Results shown are representative of three independent experiments.

Next, we determined whether downregulation of SOCS3 by miR-455-5p is also functional for NSCLC cells. Overexpression of miR-455-5p in H460 cells promoted cell proliferation, colony formation, cell migration and invasion, and tumor growth *in vivo* (Figure 4A-4D). Moreover, the pro-tumorigenic effect of miR-455-5p was inhibited when co-transfection of a miR-455-5p-resistant form of SOCS3 (Figure 4A-4D). These results suggest that targeting SOCS3 is helpful for pro-tumorigenic function of miR-455-5p.

### SOCS3 is significantly down-regulated and is correlated to poor clinical outcomes

**Figure 5:**
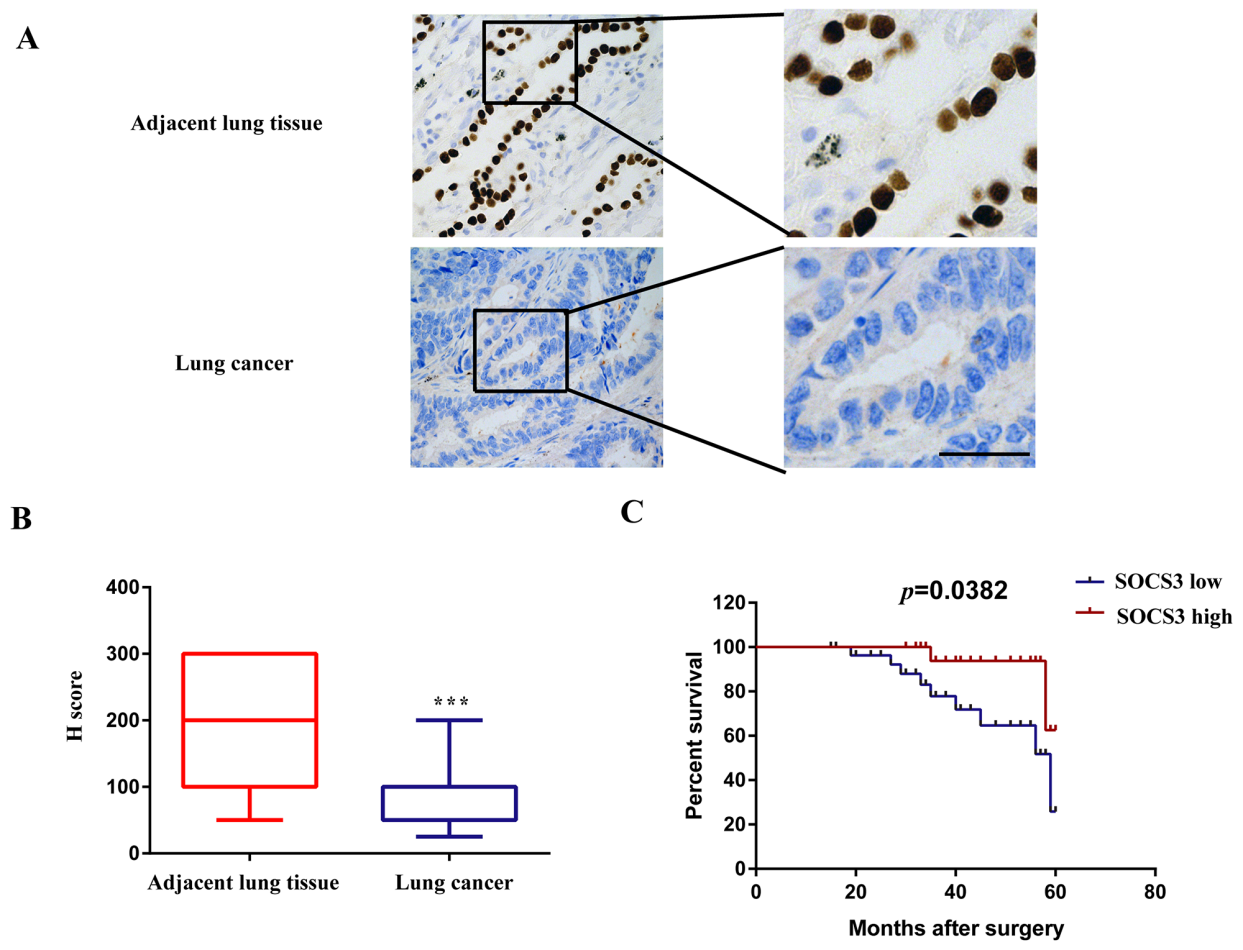
SOCS3 is significantly down-regulated in NSCLC tissues and is correlated with poor clinical outcomes **(A)** Representative immunostaining of SOCS3 expression in human NSCLC tissues and corresponding adjacent lung tissues. Bar=200μm. **(B)** Quantitative analysis of SOCS3 expression in 57 cases of paired NSCLC tissues and their corresponding adjacent lung tissues. The P-value corresponds to the comparison of SOCS3 expression between the NSCLC tissues and corresponding adjacent lung tissues. ^***^, P < 0.001. **(C)** Kaplan-Meier analysis of overall survival in all NSCLC patients according to SOCS3 protein level.

IHC assay showed that nuclear SOCS3 was strongly expressed in most non-cancerous lung tissues, however, SOCS3 was less found in NSCLC tissues (Figure 5A and 5B). NSCLC patients with high SOCS3 levels showed much longer median overall survival, compared with those with low SOCS3 levels (*P* < 0.05, Figure 5C).

### Inverse correlation between miR-455-5p and SOCS3 expression in NSCLC patients

**Figure 6:**
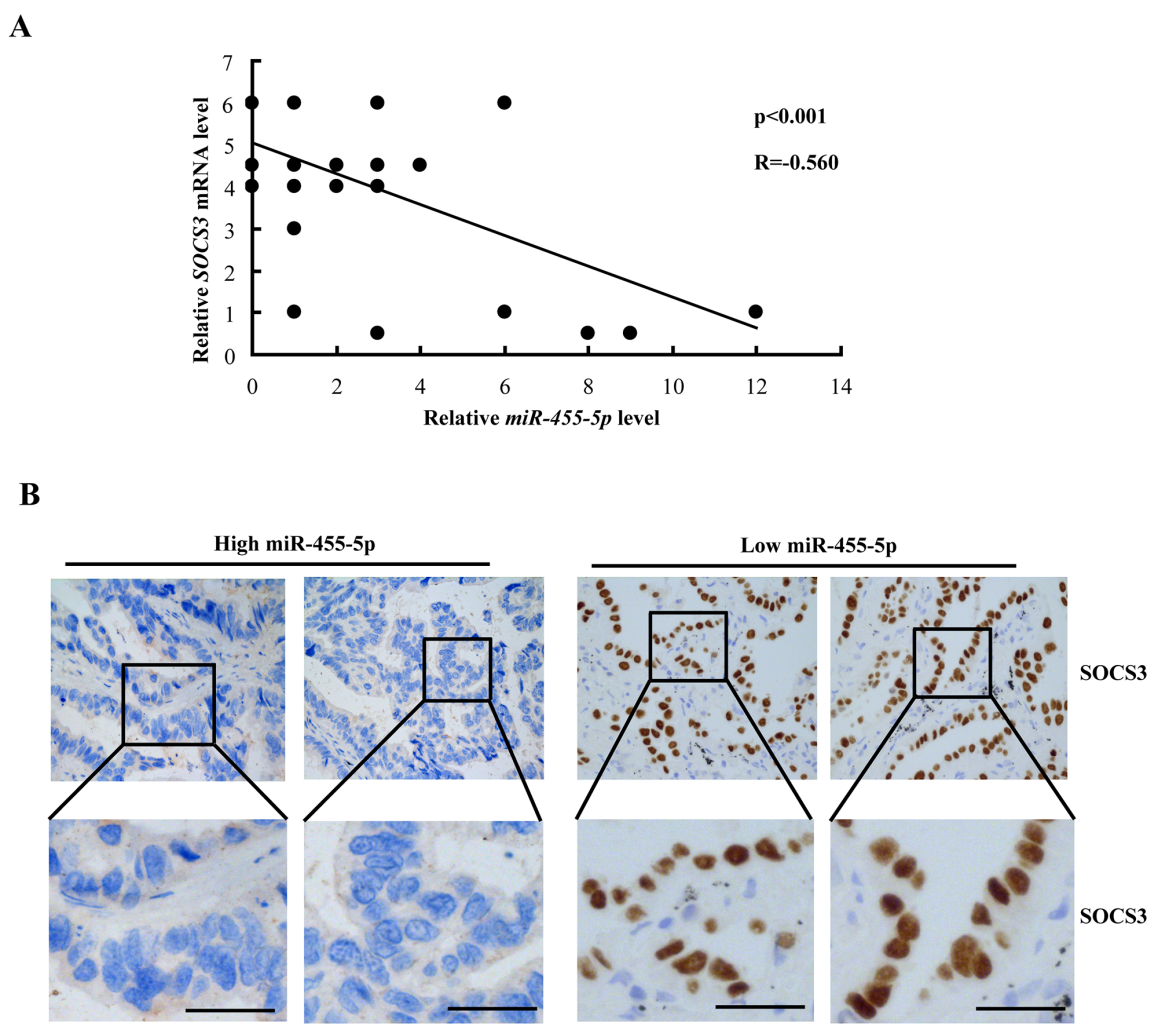
Negative correlation between miR-455-5p and SOCS3 expression in NSCLC patients **(A)** miR-455-5p and SOCS3 mRNA levels were inversely correlated in NSCLC tumors. The qRT-PCR were analyzed and shown as relative miR-455-5p levels of the Ct (cycle threshold) values, which were then converted as fold change. **(B)** representative images of SOCS3 immunohistochemical staining in NSCLC tumors with high (left) or low (right) miR-455-5p levels. Bar=200μm.

By Pearson correlation analysis, we further found there is a significant negative correlation between miR-455-5p and SOCS3 mRNA levels in tumor tissues (Figure 6A). Consistently, high miR-455-5p levels in NSCLC tumors displayed weak SOCS3 IHC staining (Figure 6B, left), however, low miR-455-5p expression displayed strong SOCS3 staining (Figure 6B, right). These results suggest that there is an inverse correlation in miR-455-5p and SOCS3.

### ERK signaling pathway promotes miR-455-5p expression

**Figure 7:**
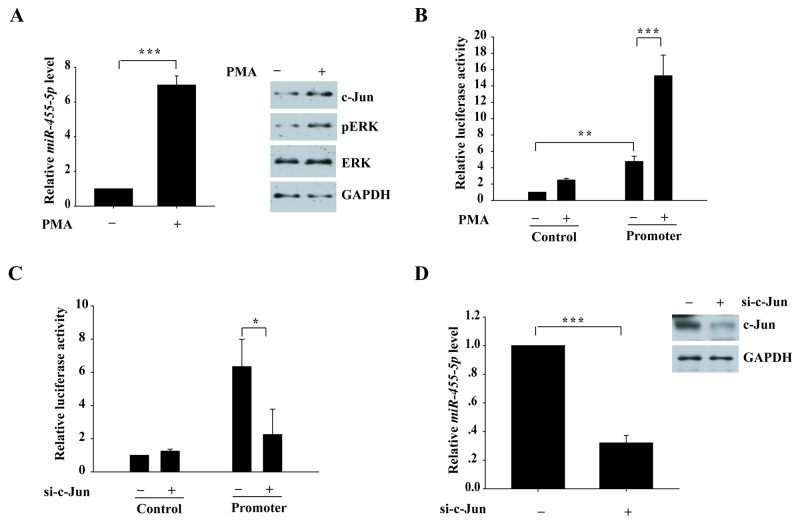
Activation of ERK signaling pathway induces miR-455-5p expression **(A)** quantitative RT-PCR for H460 cells with and without PMA treatment to measure miR-455-5p expression.^***^, P < 0.001. **(B)** Luciferase reporter construct-containing promoter region of miR-455-5p and control vector were transfected into 293T cells and treated with PMA for 24 h. ^**^, P < 0.01; ^***^, P < 0.001. **(C)** Luciferase reporter constructs containing miR-455-5p promoter and control vector were cotransfected with c-Jun siRNA into the 293T cells. ^*^, P < 0.05. **(D)** quantitative RT-PCR for H460 cells with and without c-Jun knockdown to measure miR-455-5p expression. ^***^, P < 0.001.

Next, we tested whether ERK signaling regulates miR-455-5 expression. Phorbol 12-myristate 13-acetate (PMA) in H460 cells was used to activate ERK1/2. The expression of miR-455-5p was significantly up-regulated (7 fold) with PMA stimulated compared to the control (Figure 7A). PMA treatment increased pERK and c-Jun expressions at protein level (Figure 7A). Additionally, a luciferase reporter including miR-455-5p promoter region significantly up-regulated luciferase activity (5 fold) compared to the control vector, and the effect was much stronger with PMA treatment (3 fold; Figure 7B), suggesting the ERK signaling pathway might regulate miR-455-5p. To explore whether c-Jun plays a role in the transcription of miR-455-5p, we found that knockdown of c-Jun notably inhibited miR-455-5p promoter vector-induced luciferase activity (3 folds; Figure 7C). Furthermore, knockdown of c-Jun decreased miR-455-5p expression in H460 cells (3.3 fold; Figure 7D). These results suggest that ERK signaling pathway promotes miR-455-5p expression.

## DISCUSSION

Lung cancer is very common lethal types of cancer [20–22]. It is very valuable to further explore the potential mechanisms of NSCLC due to its poor clinical outcome [23–25]. Recently, many reports have indicated that many miRNAs play key roles in tumor progression, including lung cancer [26–36].

In this study, we demonstrated that the miR-455-5p expression is significantly up-regulated in NSCLC patient samples. Serval reports have showed that miR-455-5p is abnormal expression in cancers. For example, Sand et al. showed that miR-455-5p was significantly increased in basal cell carcinoma (BCC) of the skin [13]. Shoshan et al. reported that miR-455 overexpression leads to increased melanoma tumor growth and metastasis [16]. Boisen et al. found that lower miR-455-5p expression was predictive of improved outcomes in metastatic colorectal cancer [37]. We found that miR-455-5p is an oncogene in lung caner. miR-455-5p significantly promoted tumor growth in the subcutaneous xenograft model. Interestingly, Li et al found that miR-455 was dramatically down-regulated by using NSCLC cell lines and tissues. Furthermore, miR-455 suppressed the proliferation, migration, and invasion through targeting ZEB1 in NSCLC [38]. Because a miRNA precursor generally induces two major miRNA groups, miRNA-5p and miRNA-3p, we speculated that miR-455-5p is a tumor promoter and miR-455-3p is a tumor suppressor. Indeed, miR-455-3p has been reported as a tumor suppressor [39, 40]. Taken together, our results suggest that miR-455-5p functioned as an oncogene in NSCLC.

The JAK-STAT pathway plays critical roles in the initiation and development of tumors. The SOCS3 protein is an inhibitor of activation of the JAK-STAT pathway [41, 42]. SOCS3 inhibited tumor cell proliferation, migration, and invasion in many tumor types [17–19]. We further found c-Jun binds to the miR-455-5p promoter region. Luciferase reporter assays showed that ERK activation markedly promoted the luciferase activity of a miR-455-5p promoter reporter. Accordingly, our *in vitro* analyses demonstrated increased miR-455-5p expression after activation of ERK in lung cancer cells. Overall, our results suggest that up-regulated expression of miR-455-5p might be attributable to the binding of ERK to the miR-455-5p promoter.

Overall, miR-455-5p promotes the growth, migration, invasion through directly targeting SOCS3. We further show that ERK signaling controls the regulation of miR-455-5p expression in NSCLC. Hence, the ERK/miR-455-5p/SOCS3 pathway might be an ideal target for therapeutic intervention in certain NSCLC patients.

## MATERIALS AND METHODS

### Ethics statement

Patient information and samples were obtained with written informed consent. Each patient in this study gave written informed consent to publish these case details. The research was approved by the ethics committee of Harbin Medical University Cancer Hospital.

### Clinical NSCLC tissue samples

Lung cancer specimens and matched adjacent non-cancerous tissues (n = 79) were collected from patients with NSCLC in Harbin Medical University Cancer Hospital from 2009 to 2012. The tissues were stored at −80°C until use. All samples were from patients who had not undergone preoperative radiotherapy or chemotherapy. The pathological staging of the 79 tumors was performed according to the tumor-node-metastasis (TNM) staging system.

### Cell lines

Human NSCLC cell lines, including H460, SK-MES-1, SPCA-1, A549, and H1299, the normal lung cell line 16HBE and the human embryonic kidney cell line 293T were purchased from American Type Culture Collection (ATCC) and maintained in DMEM supplemented with 10% fetal bovine serum (FBS) (Invitrogen) containing 100 units/ml penicillin and 100 units/ml streptomycin (Sigma) at 37°C with 5% CO_2_.

### Transfection

Hsa-miR-455-5p mimics and a hsa-miR-455-5p inhibitor were purchased from Sigma-Aldrich. SOCS3 siRNA was obtained from Life Technologies. H460 and H1299 cells were transfected using X-tremeGENE (Roche Applied Science), and HEK293T cells were transfected using Lipofectamine 2000 (Invitrogen) according to the manufacturer’s directions. SOCS3 siRNA was transfected into cells for 24 hours using Lipofectamine RNAiMAX Reagent (Invitrogen).

### RNA preparation and quantitative real-time PCR

Total RNAs were obtained using the TRIzol reagent (Invitrogen). Reverse transcription of RNA was performed using the ImProm-II reverse transcription system (Promega) according to the manufacturer’s instructions.

Quantitative real-time PCR was used to precisely quantify miR-455-5p expression in human NSCLC tissues or cell lines. Quantitative RT-PCR was performed with SYBR Green reagents (Takara, Japan) in a 7500 real-time PCR system from Applied Biosystems. The 2^−ΔΔCT^ method was used to measure the miR-455-5p gene expression compared with the endogenous controls (U6 non-coding small nuclear RNA). All primers for miR-455-5p and the U6 genes were designed by Primer Premier 5.0 and synthesized by Shanghai GenePharma.

### Protein extraction and western blot analysis

Cellular proteins were extracted from cultured cells with lysis buffer (50 mM Tris, pH 8.0, 150 mM NaCl, 5 mM EDTA, 50 mM NaF, and 0.1% NP-40). Protein samples were separated in sodium dodecyl sulfate (SDS)-PAGE and transferred to nitrocellulose filter membranes (Millipore, USA). After blocking in phosphate buffered saline (PBS)/Tween-20 containing 5% nonfat milk, the membranes were incubated with the following primary antibodies: SOCO3 (Santa Cruz Biotech), phosphor-ERK1/2(Cell Signaling Technology), c-Jun (Cell Signaling Technology), GAPDH (Santa Cruz Biotech). Subsequent visualization was detected with Super Signal West Femto Maximum Sensitivity Substrate (Thermo, Japan).

### Dual-luciferase reporter assay

The 3′-untranslated region (UTR) WT and mutant of human SOCS3 were amplified from human genomic DNA and individually inserted into the pmiR-RB-REPORTTM (Ribobio, Guangzhou, China). For reporter assays, cells were co-transfected with wild-type (mutant) reporter plasmid and miR-RiboTM mimics (miR-RiboTM negative control) using Lipofectamine 2000 (Invitrogen). The cells were then harvested and analyzed with the Dual Luciferase Reporter Assay system (Promega). All assays were performed in triplicate, and all values were normalized for transfection efficiency against Renilla luciferase activities. Primers are provided in Supplementary Table 1.

### *In vitro* cell proliferation assays

For cell proliferation assays, cells were seeded into each well of a 96-well plate (2000 per well) and the cell proliferation ability was determined using the Cell Counting Kit-8 (CCK8) Assay Kit (Dojindo Corp, Japan): 10 μl of the kit reagent dissolved with 100 μl DMEM was added to each well, and 2 h later the absorbance was measured at 570 nm to calculate the number of cells.

### Colony formation assay

Cells were seeded at approximately 2000 cells/well in a 6-well plate. After 14 days incubation, the cells were washed with PBS twice, fixed with methanol for 10 min, and stained with 0.5% crystal violet for 20 min at room temperature. The visible colonies were counted.

### *In vitro* transwell migration and invasion assays

Cell migration and invasion assays were performed using a 24-well plate with 8-μm pore size chamber inserts (Corning). For migration assays, 5×10^4^ cells were placed into the upper chamber per well with the non-coated membrane. For invasion assays, 1×10^5^ cells were placed into the upper chamber per well with a Matrigel-coated membrane that was diluted with serum-free culture medium. In both assays, cells were suspended in 200 μl of DMEM without FBS when they were seeded into the upper chamber. In the lower chamber, 800 μl of DMEM supplemented with 10% FBS was added. After incubation for 16 h at 37°C and 5% CO_2_, the membrane inserts were removed from the plate, and non-migrated or non-invading cells were removed from the upper surface of the membrane. Cells that moved to the bottom surface of the chamber were fixed with 100% methanol for 20 min and stained with 0.1% crystal violet for 30 min. Then, the cells were imaged and counted in at least 8 random fields. The assays were conducted three independent times.

### Immunohistochemistry

Immunohistochemistry of patient tissue sections was performed as recently described [20]. The dewaxed 5-μm sections were subjected to an antigen-demasking procedure of brief, high temperature heating of the sections immersed in citrate buffer. Endogenous peroxidases were blocked with 0.3% hydrogen peroxide, and nonspecific binding was blocked with 5% normal goat serum and 2% BSA in phosphate-buffered saline (PBS). Sections were then incubated for 2 hours at room temperature with anti-SOCO3 antibody (1:50; Santa Cruz Biotech). After washing with PBS, sections were incubated with biotinylated secondary antibody, followed by a further incubation with the streptavidin–horseradish peroxidase complex. The sections were then immersed in DAB for 5 to 10 minutes, counterstained with 10% Mayer hematoxylin, dehydrated, and mounted in crystal mount.

### Xenograft assays in nude mice

All animal work was performed in accordance with the institutional ethical guidelines for animal experimentation. Twenty-four hours after transfection of RNA oligonucleotide and/or plasmid DNA, approximately 2 × 10^6^ cells were suspended in 100 mL of DMEM and then injected subcutaneously into male BALB/c athymic nude mice at 6 to 8 weeks of age. Six mice were included in each experimental group. Tumor growth rates were examined every 5 days for 25 days. Tumor growth rates were analyzed by measuring tumor length (L) and width (W) and calculating the volume with the formula *LW*^2^/2.

### Statistical analysis

All results were presented as the mean ± standard error of the mean (SEM). A Student *t* test was performed to compare the differences between treated groups relative to their paired controls. One-way ANOVA was used to analyze tumor growth data. *P* values are indicated in the text and figures above the two groups compared with a value <0.05 (denoted by asterisks) considered significant.

## SUPPLEMENTARY FIGURES AND TABLE


