# Supplementary material for: miR-455-5p promotes cell growth and invasion by targeting SOCO3 in non-small cell lung cancer
Source: Oncotarget. 2017 Nov 20;8(70):114956–65. doi: 10.18632/oncotarget.22565 (PMC5777745; doi:10.18632/oncotarget.22565)
Supplement: Supplementary file 1 [file oncotarget-08-114956-s001.pdf]

miR-455-5p promotes cell growth and invasion by targeting SOCO3 in non-small cell lung cancer

SUPPLEMENTARY MATERIALS

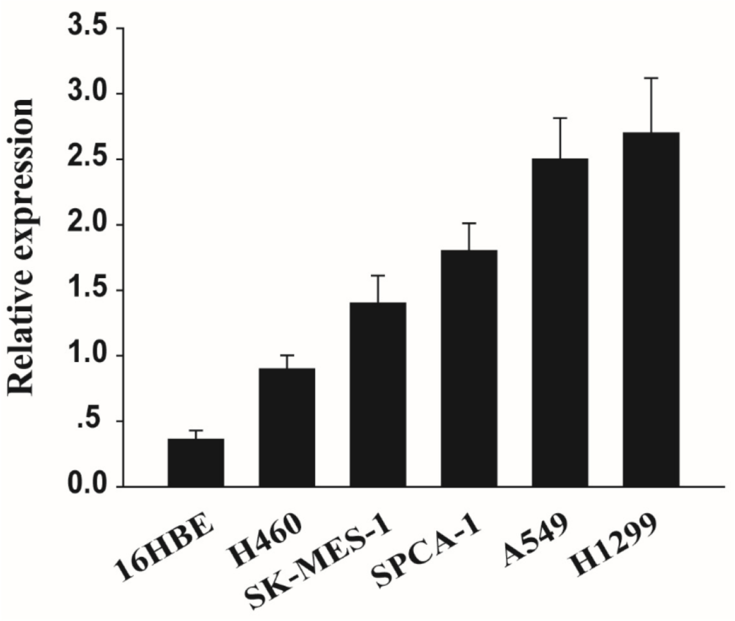

Supplementary Figure 1: The expression of miR-455-5p was measured in indicated cell lines using real-time PCR.

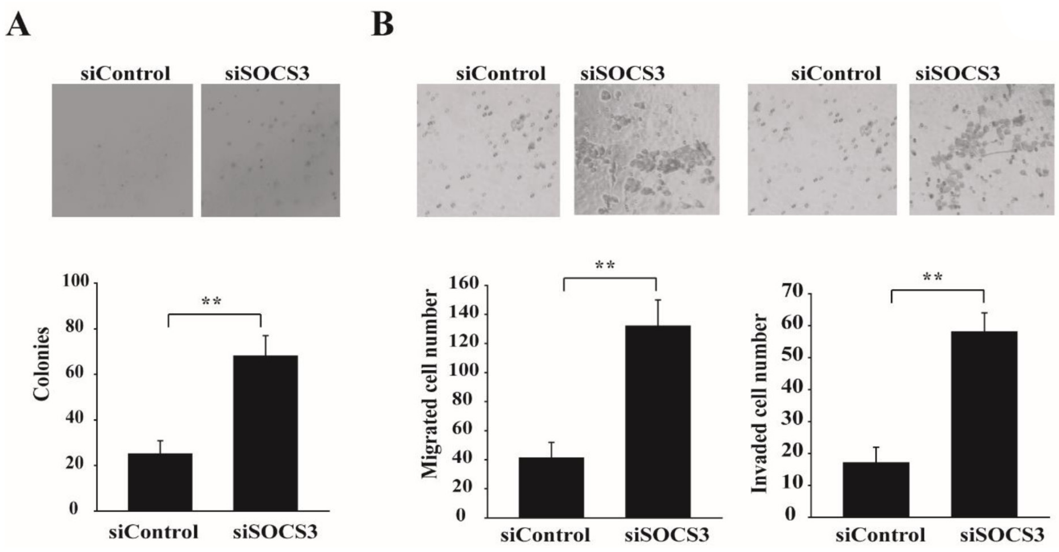

Supplementary Figure 2: (A-B) RNAi knockdown of SOCS3 in H460 cells promoted the soft-agar colony formation assays (A), Transwell cell migration and invasion (B). Error bars represent SD. **, P < 0.01. Results shown are representative of three independent experiments.

Supplementary Table 1: Primers for plasmid construction

Name		Sequence
**Plasmid construction**		
miR-445-5p	F	GCCGCCTATGTGCCTTTGGACT
	R	GTGCAGGGTCCGAGGT
U6	F	GCTTCGGCAGCACATATACTAAAAT
	R	CGCTTCACGAATTTGCGTGTCAT
SOCS3 3’UTR wt	F	TCGAGGGACGCAGGCCCCTCTCCTCCGTGGCACATGGCACAAGCACAAGAGC
	R	GGCCGCTCTTGTGCTTGTGCCATGTGCCACGGAGGAGAGGGGCCTGCGTCCC
SOCS 3’UTR mut	F	TCGAGGGACGCAGGCCCCTCTCCTCCGTCCCGCGTGGCACAAGCACAAGAGC
	R	GGCCGCTCTTGTGCTTGTGCCACGCGGGACGGAGGAGAGGGGCCTGCGTCCC
